# Effects of electroacupuncture combined with computer-based cognitive rehabilitation on mild cognitive impairment: study protocol for a pilot randomized controlled trial

**DOI:** 10.1186/s13063-019-3599-6

**Published:** 2019-08-05

**Authors:** Jae-Hong Kim, Jae-Young Han, Gwang-Cheon Park, Jeong-Soon Lee

**Affiliations:** 1 Department of Acupuncture and Moxibustion Medicine, College of Korean Medicine, DongShin University, Naju City, 58245 Republic of Korea; 2 Department of Physical and Rehabilitation Medicine, Chonnam National University Medical School and Hospital, Gwangju City, 61469 Republic of Korea; 3 Clinical Research Center, DongShin University Gwangju Korean Medicine Hospital, 141, Wolsan-ro, Nam-gu, Gwangju City, 61619 Republic of Korea; 4 Department of Nursing, Christian College of Nursing, Gwangju City, 61662 Republic of Korea; 5 Department of Acupuncture and Moxibustion Medicine, DongShin University Gwangju Korean Medicine Hospital, 141, Wolsan-ro, Nam-gu, Gwangju City, 61619 Republic of Korea

**Keywords:** Mild cognitive impairment, Electroacupuncture, Computer-based cognitive rehabilitation, Randomized controlled trial, Study protocol

## Abstract

**Background:**

Mild cognitive impairment (MCI) is defined as an intermediate stage between normal aging and Alzheimer’s disease (AD), and early and easily available interventions to delay the progress of MCI to AD are necessary. Feasible complementary and alternative therapies such as electroacupuncture (EA), exercise, and cognitive training have shown some beneficial effects on MCI and AD. Here we report the protocol for a randomized controlled trial of the efficacy and safety of EA combined with computer-based cognitive rehabilitation (EA-CCR) for the treatment of MCI.

**Methods:**

The study will be a prospective, outcome assessor-blinded, parallel-arm, single-center (DongShin University Gwangju Korean Medicine Hospital, Republic of Korea), pilot randomized controlled clinical trial with a 1:1 allocation ratio. Participants with MCI will be randomized to a computer-based cognitive rehabilitation (CCR) or an EA-CCR group (*n* = 18 each). The CCR group will receive RehaCom cognitive rehabilitation once (30 min) a day, 3 days per week (excluding Saturday and Sunday) for 8 weeks. The EA-CCR group will receive EA at Baihui (GV20), Sishencong (EX-HN1), Fengchi (GB20), and Shenting (GV24) in addition to RehaCom cognitive rehabilitation once (EA:30 min, CCR:30 min) a day, 3 days per week (excluding Saturday and Sunday) for 8 weeks. The primary outcome will be an improvement in cognitive function assessed using the Korean version of the Alzheimer’s Disease Assessment Scale-cognitive subscale. Scores for the Korean version of the Montreal Cognitive Assessment scale, Center for Epidemiological Studies Depression Scale, Korean Activities of Daily Living scale, Korean Instrumental Activities of Daily Living scale, and European Quality of Life Five Dimension Five Level Scale will be recorded as secondary outcome measures. All scores will be recorded at baseline (before intervention), 8 weeks after the first intervention (i.e., at the end of the intervention), and 12 weeks after completion of the intervention.

**Discussion:**

The study is expected to provide preliminary evidence regarding the efficacy, safety, and usefulness of EA-CCR for the treatment of MCI.

**Trial registration:**

Korea Clinical Information Service, cris.nih.go.kr, KCT0003415. Registered on 4 January 2019. Retrospectively registered, http://cris.nih.go.kr.

**Electronic supplementary material:**

The online version of this article (10.1186/s13063-019-3599-6) contains supplementary material, which is available to authorized users.

## Background

Mild cognitive impairment (MCI) is defined as a slight impairment in cognitive function (typically memory) with otherwise normal function in the performance of activities of daily living. It is now widely accepted that MCI is a transitional phase between normal function and Alzheimer’s disease (AD), during which cognitive impairment is progressing [1, 2]. Population-based studies have found that the prevalence of MCI in elderly individuals (≥ 65 years) is 10–20%, with 5–10% of patients progressing to AD each year [3–5].

The US Food and Drug Administration (FDA) has not approved any therapeutic agents that could manage symptoms in the short term or prevent/slow down the progression of MCI to AD in the long term [6]. In order to decrease the prevalence of AD, there is an urgent need for novel and effective approaches for treating MCI. Therefore, various nonpharmacological treatments such as cognitive training [7, 8], physical treatment [9], and acupuncture [10–12] have been attempted.

Acupuncture is a common traditional Chinese medicine technique used for the treatment of neuropathy, with manual and electrical stimulation of acupoints being commonly used stimulation modes [13]. Recent studies have reported that acupuncture may be an effective adjunctive therapy for neurological diseases, including stroke [14], AD [15], MCI [10–12, 16], and vascular dementia [17, 18]. The potential mechanisms of action underlying its putative effects in patients with cognitive impairment include enhanced release of neurotrophic factors, decreased oxidative damage and expression of apoptosis-related genes, improved synaptic plasticity, and proliferation and survival of neuronal precursor cells in the hippocampal CA1 and dentate gyrus area [19–21]. Computer-based cognitive rehabilitation (CCR) has been widely used since its introduction in 1986, and it has been further improved with neuroscientific and technological developments [22, 23]. CCR interventions may generate some positive effects in patients with MCI and/or dementia, such as an improvement in learning and short-term memory and amelioration of behavioral symptoms [8]. RehaCom (Hasomed Inc., Magdeburg, Germany, http://www.hasomed.de) is a software package that has been translated into different languages, including Korean. This software includes five different therapeutic programs that seek to restore attention, memory, executive functions, and the visual field. Each program has one to four different tasks from which participants can choose during each therapy session. Because RehaCom provides a battery of standardized tasks with immediate feedback, it is useful for follow-up examinations and clinical studies as well as the treatment of MCI [23].

Although electroacupuncture (EA) is used for the treatment of MCI in Korean medicine, evidence regarding its efficacy is insufficient. Here we describe the protocol for an outcome assessor-blinded, parallel-arm, pilot randomized controlled clinical trial of the efficacy and safety of EA combined with CCR (EA-CCR) for the treatment of MCI. The results of the study are expected to provide preliminary evidence regarding the usefulness of EA-CCR for patients with MCI.

## Methods/design

### Objective

This study will investigate the efficacy of EA-CCR for the treatment of MCI through a comparison of the effects of EA-CCR with those of CCR in terms of an improvement in cognitive function in patients with MCI.

### Hypothesis

The null hypothesis is that the improvement in cognitive function achieved with EA-CCR is not superior to that achieved with CCR in patients with MCI.

### Study design

The study design is in accordance with the Standard Protocol Items: Recommendations for Interventional Trials (SPIRIT) and Consolidated Standards of Reporting Trials (CONSORT) 2010 guidelines [24, 25] (see Additional file 1). The study is a prospective, outcome assessor-blinded, parallel-arm, single-center (DongShin University Gwangju Korean Medicine Hospital, Republic of Korea), pilot randomized controlled clinical trial with a 1:1 allocation ratio. A total of 36 participants who meet the inclusion and exclusion criteria will be randomly allocated to an EA-CCR or a CCR (*n* = 18 each) group. Participants in the CCR group will receive RehaCom cognitive rehabilitation only, while those in the EA-CCR group will receive EA at Baihui (GV20), Sishencong (EX-HN1), Fengchi (GB20), and Shenting (GV24) along with RehaCom cognitive rehabilitation. The treatment duration will be 8 weeks in both groups. The primary outcome measure will be an improvement in cognitive function evaluated using the Korean version of the Alzheimer’s Disease Assessment Scale-cognitive subscale (ADAS-K-cog). The secondary outcome measures will include changes in scores for the Korean version of the Montreal Cognitive Assessment (MoCA-K) scale, Center for Epidemiological Studies-Depression Scale (CES-D), Korean Activities of Daily Living (K-ADL) scale, Korean Instrumental Activities of Daily Living (K-IADL) scale, and European Quality of Life Five Dimension Five Level Scale (EQ-5D-5 L). All scale scores will be recorded at baseline (before intervention), 8 weeks after the first intervention (i.e., at the end of the intervention), and 12 weeks after completion of the intervention.

**Fig. 1:**
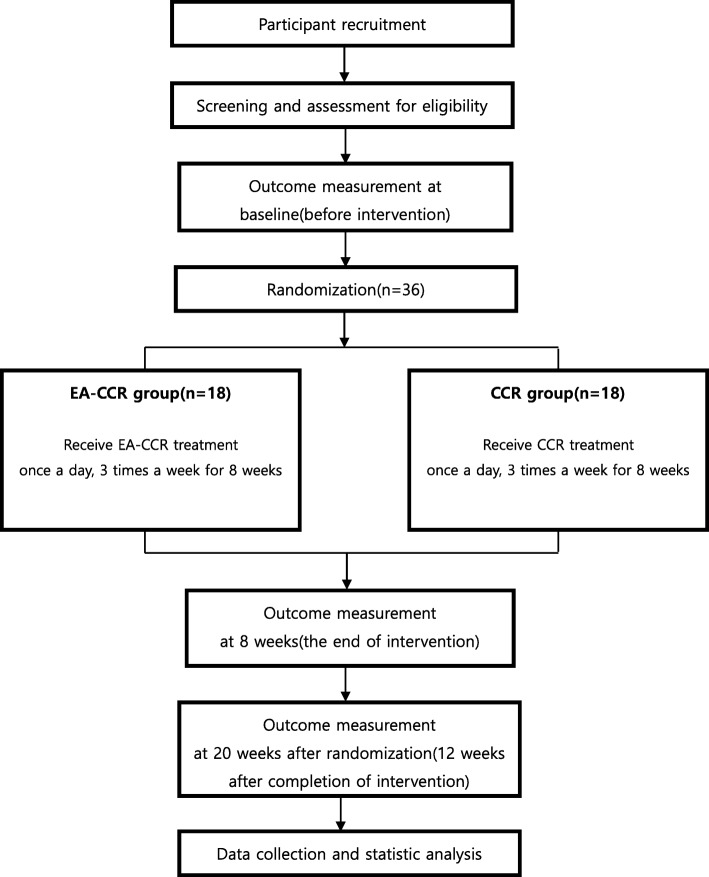
Study design flow chart

**Fig. 2:**
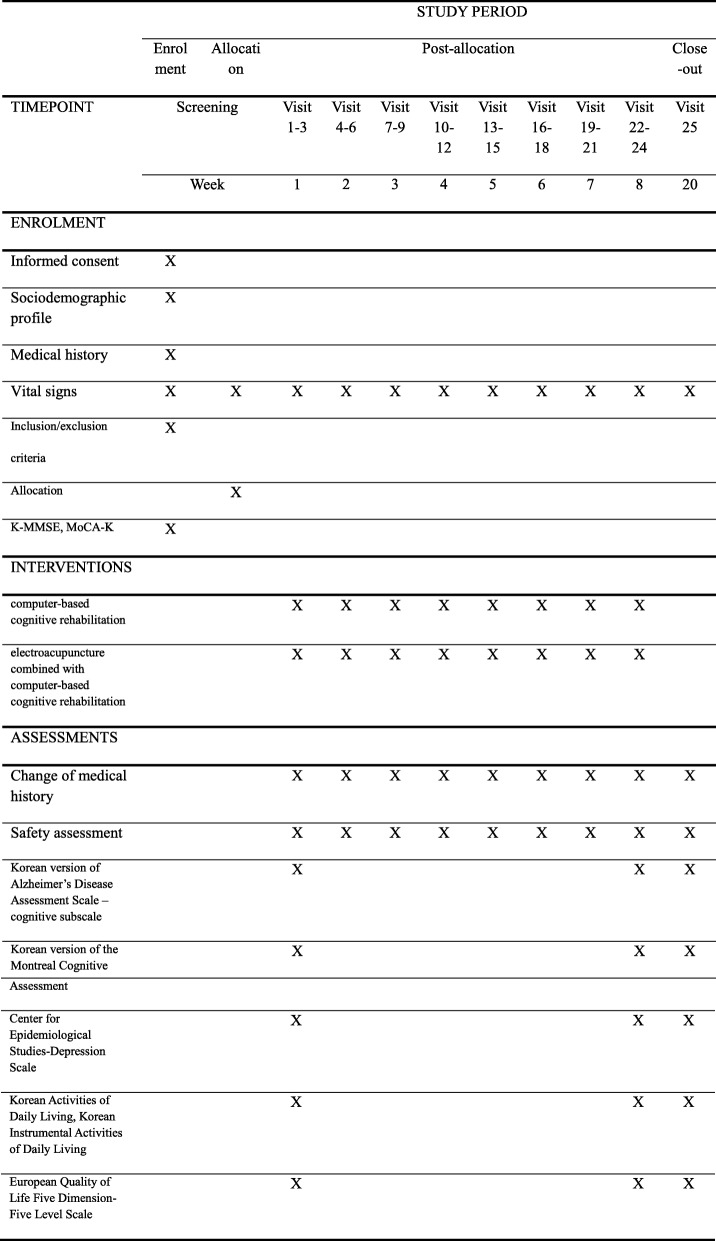
Standard Protocol Items: Recommendations for Interventional Trials (SPIRIT) figure showing the schedule of enrollment, interventions, and data collection

This study protocol complies with the principles of the Declaration of Helsinki and Korean Good Clinical Practice guidelines and has been approved by the Ministry of Food and Drug Safety (Medical Device Clinical Trial Plan approval number 859). The trial has been registered at cris.nih.go.kr (registration number, KCT0003415). The study design is summarized in Figs. 1 and 2.

### Participant recruitment

Participants will be recruited at DongShin University Gwangju Korean Medicine Hospital, Republic of Korea. The study will be advertised through local newspapers, the Internet, and posters in communities and hospitals. Individuals can enquire about participation through the telephone or personal visits. When interested individuals visit the clinical research center at DongShin University Gwangju Korean Medicine Hospital, they will receive an explanation about the study from the clinical research coordinator (CRC) and will be requested to voluntarily sign an informed consent form before participation. All recruited individuals will be screened by the Korean version of the Mini-Mental State Examination (K-MMSE) and MoCA-K for confirmation that all inclusion criteria are met. The CRC will continuously monitor the medical condition of the enrolled participants to ensure adherence to the intervention protocols.

### Inclusion criteria

Participants meeting all of the following criteria will be included in the study: (1) age 55 to 85 years; (2) fulfillment of the Petersen diagnostic criteria for MCI [1, 2] with memory impairment for at least 3 months, (3) K-MMSE score of 20–23, (4) MoCA-K scale score of 0–22, (5) Korean language fluency that is adequate for reliable completion of all study assessments, and (6) voluntary provision of informed consent.

### Exclusion criteria

The exclusion criteria are as follows: (1) diagnosis of dementia according to the Diagnostic and Statistical Manual of Mental Disorders-IV; (2) history of structural brain lesions that can cause cognitive impairment, such as traumatic brain injury, stroke, intracranial space-occupying lesions, and congenital mental retardation; (3) presence of cancer and/or serious cardiovascular, cerebrovascular, liver, or kidney disease; (4) history of treatment for alcohol or drug dependency or mental diseases such as schizophrenia, serious anxiety, or depression in the past 6 months; (5) ongoing treatment for MCI, such as medication, acupuncture, or cognitive training); (6) difficulties in assessment due to visual and hearing impairments; (7) presence of contraindications for EA, such as blood clotting abnormalities (e.g., hemophilia), infection of the skin over the head, or the presence of a pacemaker); and (8) concurrent participation in other clinical trials.

### Ethical considerations

This study has been approved by the institutional review board (IRB) of DongShin University Gwangju Korean Medicine Hospital, Republic of Korea. The purpose and potential risks of this clinical trial will be fully explained to the participants and their families. All participants will be asked to provide written informed consent before participation.

### Randomization

Following the acquisition of written informed consent, the practitioners who will perform the intervention will conduct a screening interview. Then, the assessor will perform baseline measurements for participants who meet the inclusion criteria. The 36 enrolled participants will be immediately assigned serial numbers generated using SPSS version 21 software (IBM Corp., Armonk, NY, USA) and randomly allocated to one of the two study groups (*n* = 18 each). The serial number codes will be inserted into opaque envelopes that will be sealed and stored in a double-locked cabinet; these will be opened by the principal investigator (PI) or practitioners, who will perform the intervention in the presence of the patient and a guardian.

### Implementation

The CRC will generate the allocation sequence, enroll participants, and assign participants to the intervention.

### Blinding

During the course of this clinical trial, the assessor will not contact any participant at any point of time other than the time of assessment. Furthermore, unblinding will not be permitted. However, if serious adverse events (SAEs) occur, unblinding will be permitted after an agreement among the researchers. For prevention of bias with regard to selection, performance, and attrition caused by nonblinding of participants and practitioners, only individuals without conflicts of interest or preconceived positions will be involved in this study. All practitioners who will perform the interventions will receive training in clinical trials before participation.

### Interventions

Participants in the CCR group will receive RehaCom cognitive rehabilitation once (30 min) a day, 3 days per week (excluding Saturday and Sunday) for 8 weeks. Participants in the EA-CCR group will receive EA at Baihui (GV20), Sishencong (EX-HN1), Fengchi (GB20), and Shenting (GV24) in addition to RehaCom cognitive rehabilitation once (EA:30 min, CCR:30 min) a day, 3 days per week (excluding Saturday and Sunday) for 8 weeks. EA will be performed first, followed by CCR. The treatment will be administered by Korean medicine doctors with 6 years of formal university training in Korean medicine and a license to administer treatment. To ensure strict adherence to the study protocol, the doctors will receive training together and use the same techniques.

#### Electroacupuncture treatment

EA will be performed at the following acupoints: Baihui (GV20), Sishencong (EX-HN1), Fengchi (GB20), and Shenting (GV24, [10]). Only sterile, stainless steel, disposable acupuncture needles (size 0.25 × 30 mm, product no. A84010.02; Dong Bang Acupuncture, Inc., Boryeong, Republic of Korea) with guide tubes and an EA stimulator [CELLMAC PLUS (STN-330), product no. A16010.04; Stratek, Co., Ltd., Anyang, Republic of Korea] will be used. With the patient in a sitting position, the needles will be subgaleally inserted at an angle of 15–30° along the scalp. GB20 will be punctured 17–30 mm in the direction of the tip of the nose. GV24, the anterior EX-HN1, and GV20 will be punctured in the forward direction, while the left, right, and posterior EX-HN1 will be punctured in the direction of GV20. The depth of insertion will be 9–24 mm depending on the location of the needle [26]. After insertion, the needles will be left in position for 30 min. Manual stimulation will not be used. GV24 and GV20, the left and right EX-HN1, the anterior and posterior EX-HN1, and the left and right GB20 will be subjected to EA under the following parameters: continuous waves, frequency 3–15 Hz, and intensity 2–4 mA. Each participant will receive a total of 24 30-min sessions (three times per week for 8 weeks [10] see (Table 1).

**Table 1:** Revised Standards for Reporting Interventions in Clinical Trials of Acupuncture (STRICTA)

	Item criteria	Description
1. Acupuncture rationale	1a) Style of acupuncture	Korean medicine therapy
	1b) Reasoning for treatment provided—based on historical context, literature sources, and/or consensus methods, with references where appropriate	1) Discussion among four doctors who practice Korean medicine (consensus) 2) Textbook of acupuncture and moxibustion medicine 3) Relevant articles [10] Selection of treatment regions based on textbooks, related papers, and expert discussions
	1c) Extent to which treatment varied	Standardized treatment
2. Details of needling	2a) Number of needle insertions per subject per session (mean and range where relevant)	8
	2b) Names (or location if no standard name) of points used (unilateral/bilateral)	Baihui (GV20), Sishencong (EX-HN1), Fengchi (GB20), and Shenting (GV24)
	2c) Depth of insertion, based on a specified unit of measurement or on a particular tissue level	After the needles are inserted into the acupoints subgaleally along the scalp at an angle of 15–30°, GB20 will be punctured 17 –30 mm in the direction toward nose tip. GV24, the anterior EX-HN1, and GV20 will be punctured forwards, and the left, right and posterior EX-HN1 toward GV20. The depth of insertion will be 9–24 mm depending on the location of the needle [26]
	2d) Responses sought	No de qi or muscle twitching; only sensation due to needle insertion
	2e) Needle stimulation	Electrical stimulation
	2f) Needle retention time	30-min per session
	2 g) Needle type	Sterile, stainless steel, disposable acupuncture needles (size 0.25 × 30 mm; Dong Bang Acupuncture, Inc., Boryeong, Republic of Korea; Product no. A84010.02)
3. Treatment regimen	3a) Number of treatment sessions	24
	3b) Frequency and duration of treatment sessions	Three times/week for 8 weeks, 30 min per session
4. Other treatment components	4a) Details of other interventions administered to the acupuncture group	RehaCom cognitive rehabilitation
	4b) Setting and context of treatment—including instructions to practitioners—as well as information and explanations given to patients	Practitioner-patient conversation about the context of the treatment, life habits, and daily life management
5. Practitioner background	5a) Description of participating acupuncturists	Korean medicine doctor with the following qualifications: 6 years of formal university training in Korean medicine, a license
6. Control or comparator interventions	6a) Rationale for the control or comparator in the context of the research question with sources that justify the choice	Zhang H, Zhao L, Yang S, Chen Z, Li Y, Peng X, Yang Y, Zhu M. Clinical observation on effect of scalp acupuncture for mild cognitive impairment [10]
	6b) Precise description of the control or comparator; details for items 1–3 above with the use of sham acupuncture or any other type of acupuncture-like control	Elctroacupuncture combined with computer-based cognitive rehabilitation group will receive the RehaCom cognitive rehabilitation after electroacupuncture treatment by same practitioner. The participants will be generally in seating position and will receive CCR using the RehaCom software. We will use 6 different therapeutic programs that seek to restore attention, memory, and executive functions. Each program has different tasks from which participants choose during each therapy session. The CCR will last 30 min each time, three times a week, for a total of 24 sessions

#### RehaCom cognitive rehabilitation

All participants will receive RehaCom cognitive rehabilitation in the sitting position. Six different therapeutic programs that seek to restore attention, memory, and executive functions will be used. Each program has one to four different tasks from which participants can choose during each therapy session. Each participant will receive a total of 24 30-min sessions (three times per week for 8 weeks).

During the clinical trial period, all participants will be allowed to use routine management regimens, existing medications (e.g., those for hypertension, diabetes, or hyperlipidemia), and medications for maintaining and improving their health status. However, they will not be permitted to engage in other treatments for ameliorating MCI symptoms. All medical devices, including the acupuncture needles, EA stimulator, and RehaCom software, will be inspected by the investigators, who will record the results of check-ups in the management register.

### Outcome measurements

Scores for the ADAS-K-cog, MoCA-K scale, CES-D, K-ADL scale, K-IADL scale, and EQ-5D-5 L will be recorded before treatment, at the end of treatment, and at 12 weeks after treatment completion.

#### Primary outcome

In accordance with the study objective, an improvement in cognitive function assessed using the ADAS-K-cog will be considered the primary outcome of the study. The ADAS-cog was developed by Rosen et al. [27] for the evaluation of comprehensive cognitive functions involving memory, language, praxis, and frontal lobe function. It was subsequently translated into Korean and validated [28]. This tool is sensitive to changes over time and includes subscales for testing list learning, object naming, commands, ideational apraxia, construction, orientation, and recognition. The possible total score is 70, with a higher score indicating a higher degree of deficit [29].

#### Secondary outcomes

The secondary outcomes will include changes in the MoCA-K scale, CES-D, K-ADL scale, K-IADL scale, and EQ-5D-5 L scores over time.

The MoCA scale, which was developed by Nasreddine et al., is a clinician-friendly, validated, brief instrument with high sensitivity and specificity for the detection of MCI [30]. It has been translated into Korean and validated [31].

The CES-D is a widely used 20-item self-report instrument designed to measure depressed affect, positive affect, somatic and retarded activity, and interpersonal relationships. Patients are asked to rate each item using an ordinal 4-point Likert scale [32].

The K-ADL and K-IADL scales will be used to assess physical function. The K-ADL scale was developed to assess basic activities of the elderly, including dressing, washing, bathing/showering, eating, getting out of bed/room, using the toilet, and controlling urination. The K-IADL scale is used to estimate more complex activities necessary for independent daily life. These include personal grooming, household chores, preparing meals, doing laundry, going out within a short distance, using transportation, shopping, managing money, making phone calls, and taking medications [33]. A combination of ADL and IADL items in a single scale would provide an enhanced measurement range and sensitivity [34].

The European Quality of Life Five Dimension Scale (EQ-5D) is a generic instrument for assessment of the health-related quality of life. It is based on a descriptive system that defines health in terms of five dimensions: mobility, self-care, usual activities, pain/discomfort, and anxiety/depression. Each dimension has three response categories: no, some, or extreme problems. The EQ-5D-5 L, which will be used in this study, is a new version of the EQ-5D that includes five levels of severity in each of the existing five dimensions [35].

### Incidence of adverse events

Adverse events (AEs) are undesirable and unintentional signs, symptoms, or diseases that appear during or after treatment in a clinical trial. The participants in this study will be required to voluntarily report any AEs. All AEs that occur during the trial will be documented. Possible AEs include skin irritation; bleeding; local hematoma; pallor, sweating or dizziness; fainting during EA treatment; needle retention after treatment; continuous severe pain for > 1 h after EA; objective worsening of existing symptoms; and undesirable and unintentional signs, symptoms, or diseases. The CRC will record all AEs in detail, including the time and date of occurrence, degree of severity, any measures related to treatment of the AE, and any potentially causal relationship between the treatment and the AE, and will report all AEs to the PI and relevant IRB. In case of SAEs, defined as those causing severe disability or malfunction, appropriate measures will be taken, and the incident will be immediately reported to the PI and relevant IRB. In case an AE occurs because of the clinical trial, participants will notify the CRC and PI and will be compensated.

### Quality assurance

This protocol has been reviewed and revised several times by experts on acupuncture, rehabilitation, neurology, statistics, and methodology. Before the trial, all researchers will be required to attend a series of training sessions which will ensure that the involved personnel fully understand the trial protocol and standard operating procedures (SOPs) that will be employed during the study. The Data Monitoring Committee will comprise the PI and CRC. The clinical trial will be monitored by a clinical research associate (CRA) who will check all documents related to the clinical trial, including the case report forms (CRFs) and SOPs, and ensure that the clinical trial is conducted in accordance with the prescribed protocols and SOPs. Monitoring will be carried out by an independent CRA who will not be involved in any other aspect of the trial. In the event that the protocol described herein is revised, the revisions will require approval from the Ministry of Food and Drug Safety and the IRB of DongShin University Gwangju Korean Medicine Hospital.

### Sample size estimation

Because of the lack of adequate preliminary studies and limited research funds, study period, and recruitment opportunities, we have adopted a pilot study design with 18 participants in each group.

### Statistical analysis

A statistician who is not involved in the clinical trial will analyze the final data. We will perform a per-protocol analysis (PP group) for the assessment of efficacy with a supplementary full analysis set (FA group). We will compare the results of analyses between the PP and FA groups and confirm whether there are statistically significant differences between the two groups. If there is a significant difference, the cause will be reviewed and reflected in the efficacy assessment. All statistical analyses will be performed using SPSS version 21 software (IBM Corp.).

Baseline characteristics will be described and compared. Continuous data will be presented as means and standard deviations and compared using the independent *t* test or Wilcoxon rank sum test, while categorical data will be presented as frequencies and percentages and compared using the chi-squared test or Fisher’s exact test.

Changes in scores for the ADAS-K-cog, MoCA-K scale, CES-D, K-ADL scale, K-IADL scale, and EQ-5D-5 L at 8 weeks (at the end of the intervention) and 20 weeks (12 weeks after the end of the intervention), relative to the baseline score, will be analyzed for each group using the paired *t* test or Wilcoxon signed rank test. The degree of changes at each time point will be evaluated using repeated measures analysis of variance and the two-sample *t* test or Wilcoxon rank sum test. Subanalyses will be applied to the statistical analysis according to the participant age. All reported *P* values will be two-sided with confidence intervals at the 95% levels. A *P*value of < 0.05 will be considered statistically significant.

Data for participants who meet the dropout criteria (i.e., < 75% compliance with the protocol procedures [received less than 18 of the 24 scheduled treatment sessions], incidence of SAEs, reluctance to continue the trial, incomplete data that could influence the trial, large error in protocol or significant deviation in implementation, or decision to terminate trial participation by the PI or IRB) will be excluded. Missing values will be implemented by the last observation carried forward method. Interim analyses will not be performed.

### Confidentiality and data management

All identification records of the participants will be kept confidential. When the results of the study are published, the identification records can be accessed under IRB approval. All documents related to the trial, including CRFs, will be recorded and labeled with participant identification codes and will not reveal the name of the participant. These serial number codes will be stored in sealed, opaque envelopes and kept in a double-locked cabinet. All participant data will be recorded in Excel files by the CRC, and these electronic data will be stored in a password-protected computer. Individuals who are not authorized by the IRB cannot access the data. In addition, raw data (CRFs) will be stored in a cabinet until the end of the study. Written informed consent for the publication of individual details and accompanying images will be obtained from the participants.

## Discussion

The design of this study, including the treatment and evaluation schedules, is based on the designs of several studies evaluating acupuncture treatment for MCI [10, 16].

Although acupuncture has long been used in the treatment of clinical disorders, including cognitive dysfunction, its possible effects on cognitive function have received little attention, resulting in a poor evidence base. This study is expected to provide preliminary evidence for the efficacy, safety, and usefulness of EA for the treatment of MCI.

In this study, cognitive function will be assessed in the initial screening session using the K-MMSE and MoCA-K scale. The Mini-Mental State Examination (MMSE) is one of the most commonly used evaluation tools for cognitive function, while the MoCA scale is a simple and highly sensitive cognitive screening tool [36]. Changes in the ADAS-K-cog score will be recorded as the primary outcome, while changes in scores for the MoCA-K scale, CES-D, K-ADL scale, K-IADL scale, and EQ-5D-5 L will be recorded as secondary outcomes. This will aid in evaluation of the effects of EA-CCR on cognitive function and depression, activities of daily living, and quality of life in patients with MCI.

This protocol has some limitations. First, because of the lack of adequate preliminary studies and the limited research fund, the study has been designed as a single-center pilot study with a small sample size. Second, we will not be able to compare the findings with those for other acupuncture treatment methods for MCI because of the small sample size. Third, although all outcome measures will be measured and recorded by an independent researcher in order to minimize the risk of detection bias, the acupuncturists and participants cannot be blinded to the group allocation.

Nevertheless, the results of this study are expected to provide preliminary evidence regarding the usefulness, safety, and efficacy of EA-CCR for the treatment of MCI, thus providing a foundation for further research.

### Dissemination policy

We will report the final data to the Ministry of Health and Welfare through the Korea Health Industry Development Institute. We will also publish the results after study completion.

### Trial status

This trial (protocol version number EA-CCR version 1.2; approved on September 17, 2018) is ongoing. The trial was approved by the Ministry of Food and Drug Safety (approval number 859). The recruitment began on November 29, 2018 and is expected to be complete by the end of June 2020. Trial procedures are expected to be complete by the end of September 2020.

## Additional file

Additional file 1: SPIRIT 2013 checklist: recommended items to address in a clinical trial protocol and related documents. (DOCX 367 kb)

## Data Availability

Not applicable; no data have been generated as yet.

## Abbreviations

AD: Alzheimer’s disease
ADAS-K-cog: Korean version of Alzheimer’s Disease Assessment Scale-cognitive subscale
AE: Adverse event
CCR: Computer-based cognitive rehabilitation
CES-D: Center for Epidemiological Studies Depression Scale
CONSORT: Consolidated Standards of Reporting Trials
CRA: Clinical research associate
CRC: Clinical research coordinator
CRF: Case report form
EA: Electroacupuncture
EA-CCR: Electroacupuncture combined with compute-based cognitive rehabilitation
EQ-5D-5 L: European Quality of Life Five Dimension Five Level scale
IRB: Institutional review board
K-ADL: Korean Activities of Daily Living
K-IADL: Korean Instrumental Activities of Daily Living
K-MMSE: Korean version of Mini-Mental State Examination
MCI: Mild cognitive impairment
MMSE: Mini-Mental State Examination
MoCA-K: Korean version of the Montreal Cognitive Assessment
PI: Principal investigator
SAE: Serious adverse event
SOP: Standard operating procedure
SPIRIT: Standard Protocol Items: Recommendations for Interventional Trials
